# Targeting Eukaryotic Elongation Factor 1A: How Small-Molecule Inhibitors Suppress Tumor Growth via Diverse Pathways

**DOI:** 10.3390/ijms26157331

**Published:** 2025-07-29

**Authors:** Han Zhang, Siqi Yu, Ying Wang, Shanmei Wu, Changliang Shan, Weicheng Zhang

**Affiliations:** College of Pharmacy, Tianjin Key Laboratory of Molecular Drug Research, Nankai University, Tianjin 300353, China

**Keywords:** anticancer, mechanism, pathway, eEF1A, inhibitor

## Abstract

Eukaryotic elongation factor 1A (eEF1A), the second most abundant intracellular protein, not only plays a key role in peptide elongation, but is also capable of numerous moonlighting functions. Within malignant cells, eEF1A is by no means a neutral bystander but instead actively participates in oncogenic transformations via a myriad of molecular pathways. Thus far, a broad range of small-molecule inhibitors have been identified, which, despite their structural diversity, suppress tumor growth by targeting eEF1A. Interestingly, just as eEF1A enables its oncogenic potential far beyond boosting protein translation, these targeted agents disrupt this oncoprotein via multiple axes distinct from mere protein synthesis inhibition. Whereas the oncogenic mechanisms of eEF1A has been well documented, there lacks a systemic survey of the eEF1A-targeting agents in terms of their mechanisms. Accordingly, the present work aims to examine their multifaceted modes of action more than just blocking protein synthesis. By unveiling these insights, our deepened knowledge of these eEF1A-binding inhibitors will inform the development of future eEF1A-targeted drugs for cancer treatment.

## 1. Introduction

Cancer is basically a disease of dysregulated cellular homeostasis driven by the progressive accumulation of somatic mutations, which confer malignant phenotypes such as uncontrolled proliferation, tissue invasion, and metastatic dissemination [1,2]. These hallmarks emerge from the genomic instability and intratumoral heterogeneity inherent in cancerous cells, posing formidable therapeutic challenges [3]. While surgical resection remains a cornerstone for localized tumors, modern oncology employs a multifaceted arsenal including radiotherapy [4,5], chemotherapy [6], biologics [7], and breakthrough immunotherapies [8], yet significant limitations persist. Current therapies face three major challenges: (1) the inevitable development of resistance through genetic and epigenetic adaptations; (2) dose-limiting toxicities that compromise treatment efficacy and patient quality of life; and (3) limited efficacy against metastatic disease. These unmet clinical needs drive the search for alternative strategies with novel mechanisms of action. Of note, targeted therapies, in which well-defined small-molecule chemicals or biomacromolecular agents precisely attack cancer-specific vulnerabilities without causing non-selective toxicity, have revolutionized the cancer treatment paradigms [9,10]. However, clinical experience reveals that even these precision approaches are constrained by tumor evolution and microenvironmental adaptations, highlighting the need for interventions targeting the more fundamental aspects of cancer cell biology. Among these approaches, targeting translational machinery components represents an emerging frontier, which exploits cancer cells’ heightened dependence on protein synthesis for survival and adaptation [11,12,13,14].

Eukaryotic elongation factor 1A (eEF1A) is an indispensable component of the translation machinery, whose canonical function is recruiting aminoacyl-tRNAs (aa-tRNAs) to the ribosomal A site for peptide elongation [15]. It is the second most abundant cellular protein, widely distributed across cytoplasmic and nuclear compartments. There are two highly homologous yet functionally distinct isoforms of eEF1A, that is, eEF1A1 and eEF1A2, in higher vertebrates, encoded by paralogous genes *EEF1A1* (chromosome 6q14) and *EEF1A2* (chromosome 20q13.3), respectively. Despite sharing 92% amino acid sequence identity and 98% similarity, these isoforms exhibit divergent expression profiles and developmental regulation [16]. eEF1A1 is ubiquitously expressed in most tissues during embryonic development and remains the predominant isoform in non-muscle adult tissues. By comparison, eEF1A2 shows a tightly restricted expression pattern, becoming the major adult form in neurons, cardiomyocytes, and skeletal myotubes after a developmental switch that silences *EEF1A1* in these cell types [17]. The functional divergence between these two isoforms stems from subtle sequence variations that impact their biochemical properties. For example, a key difference lies in nucleotide binding kinetics, where eEF1A2 exhibits a 7-fold slower GDP dissociation rate compared to eEF1A1. This result was attributed to a single amino acid substitution (Asn197 in eEF1A1 vs. His197 in eEF1A2) [18]. Structural studies reveal distinct conformational states: eEF1A1 primarily functions as a monomer in its GTP-bound form during translation elongation, while eEF1A2 readily forms dimers in its GDP-bound state, with the dimerization interface involving conserved residues that are frequently mutated in neurodevelopmental disorders. These isoforms also differ in their post-translational modification patterns and subcellular localization. For instance, eEF1A2 undergoes unique phosphatidylethanolamine modifications at Glu301 and Glu374 that facilitate membrane association, and displays differential phosphorylation signatures that regulate its interactions with cytoskeletal components [19]. A detailed comparison of eEF1A1 and eEF1A2 Isoforms is given in Table 1. Since many studies do not differentiate between eEF1A1 and eEF1A2, the term eEF1A will be used throughout this discussion to refer collectively to both isoforms, unless a particular isoform is specified by the cited works.

Apart from assisting peptide elongation, eEF1A is endowed with numerous moonlighting functions important to cellular homeostasis [20,21]. However, moving to malignant cells, it turned out to be causally linked to oncogenesis [22,23,24]. Since the landmark study by Anand et al. [25], the dysregulated expression of eEF1A—particularly the *EEF1A2* isoform—has been reported across a wide spectrum of malignancies, including breast, ovarian, lung, liver, prostate, pancreatic, colorectal, gastric, renal, testicular, thyroid, and hematopoietic cancers, as well as certain sarcomas. In many cases, elevated eEF1A levels correlate with advanced disease stages and poor prognosis, leading to its recognition as an independent prognostic biomarker [26]. A recent study found that the overexpression of eEF1A2 correlates with disease progression in lung adenocarcinoma, with the highest expression in invasive versus preinvasive lesions. This expression pattern associates with aggressive features (vascular/pleural invasion) and poorer survival, driven in part by *EEF1A2* gene amplification [27]. In gastric cancer, eEF1A2 demonstrates tumor-specific hypomethylation and overexpression linked to the pro-tumorigenic Hedgehog signaling pathway. The persistence of these epigenetic alterations from early intramucosal gastric cancers (IMCs) to advanced stages, as confirmed by The Cancer Genome Atlas (TCGA) data, positions eEF1A2 as a progression biomarker [28].

**Figure 1:**
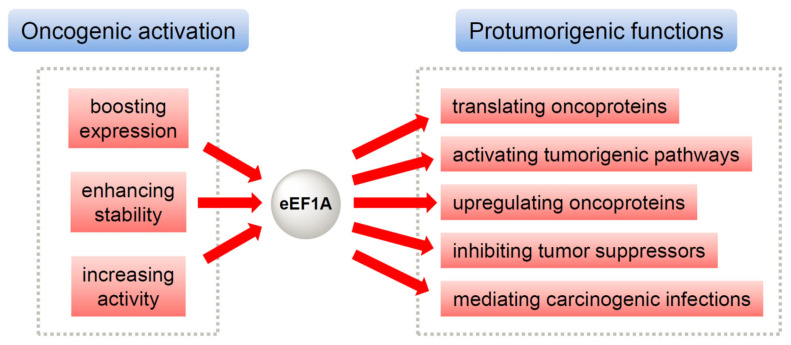
The malignant nature of eEF1A originates from (1) the oncogenic activation and/or upregulation of eEF1A per se (**left**) and (2) eEF1A-mediated activation and/or upregulation of other oncoproteins as well as protumorigenic pathways (**right**).

The mechanism responsible for eEF1A’s oncogenic properties has generated substantial research interest. Accumulating studies reveal two scenarios where eEF1A exerts its oncogenic effects (Figure 1) [26]. First, eEF1A as an oncoprotein is able to drive oncogenesis through multipronged mechanisms (Figure 1, right). Besides boosting protein translation, it upregulates other oncoproteins and key oncogenic cascades, inhibits tumor suppressors, and contributes to infection-associated cancers. These pleiotropic roles underscore eEF1A as a central node in oncogenic networks. Second, eEF1A is subject to multiple regulatory mechanisms including transcriptional upregulation, post-transcriptional stabilization, and post-translational modifications (Figure 1, left), which converge to sustain elevated eEF1A levels and functionality conducive to tumor proliferation, metastasis, and therapy resistance.

Parallel to investigations into eEF1A-mediated oncogenesis, a variety of small-molecule inhibitors (Table 2) have been discovered that exert their anticancer effects by targeting eEF1A [14]. The most famous among them are didemnin B [29] and plitidepsin (dehydrodidemnin B) [30]. Both have progressed through multiple clinical trials, and the latter eventually gained approval in Australia for the treatment of relapsed/refractory multiple myeloma [31]. Metarrestin, another eEF1A-targeting molecule, is currently undergoing a phase I clinical trial for metastatic solid tumors [32]. These milestones not only validate eEF1A as a druggable target but also highlight the translational potential of its inhibitors, demonstrating that the pharmacological modulation of eEF1A can achieve the desired clinical efficacy with manageable toxicity. To our surprise, although numerous eEF1A inhibitors have been reported and their clinical therapeutic potential is increasingly recognized, a systematic discussion of their multifaceted mechanisms remains lacking in the literature. To address this gap, the present review comprehensively evaluates the current evidence and delineates the molecular basis of eEF1A-targeting strategies. In this review, we specifically focus on those eEF1A-targeting agents whose anticancer activities have been elucidated through systematic mechanistic studies, particularly those demonstrating involvement in complex signaling networks. These compounds offer not only the direct inhibition of the eEF1A function but also the modulation of downstream oncogenic pathways, which collectively contribute to their therapeutic efficacy. In contrast, eEF1A inhibitors that have merely been reported to have exhibited antiproliferative activity but without dedicated mechanistic investigation will not be discussed in detail here. By concentrating on well-characterized compounds with elucidated molecular modes of action, this review aims to provide a deeper understanding of how eEF1A inhibition translates into anticancer effects and to offer insights into the rational design of next-generation eEF1A-targeting therapeutics.

## 2. The Mechanisms of eEF1A-Targeting Anticancer Inhibitors

### 2.1. Didemnins and Tamandarins

The didemnin family, represented by didemnin B and plitidepsin (dehydrodidemnin B), is the first class of marine-derived cyclic depsipeptides identified as potent eEF1A inhibitors with broad anticancer activity [33]. Isolated from the tunicate *Trididemnum solidum*, didemnin B was the first marine natural product to enter clinical trials, demonstrating antitumor, antiviral, and immunosuppressive effects at nanomolar concentrations. Tamandarins A and B are structural analogs of didemnins isolated from Brazilian ascidians [33]. Sharing similar biological activities as didemnins, they are believed to act through the same eEF1A-targeting mechanism. While the clinical trials of didemnin B were discontinued due to toxicity, its close analog plitidepsin has shown improved safety and efficacy, leading to approval in Australia for relapsed/refractory multiple myeloma [31].

The didemnin family, including didemnin B and its clinically approved analog plitidepsin, exerts potent anticancer effects primarily through high-affinity binding to the GTPase domain of eEF1A (Domains I-II), where its depsipeptide macrocycle engages key conserved residues of eEF1A, a key regulator of protein synthesis and cellular homeostasis [50]. By stabilizing the GTP-bound conformation of eEF1A, these compounds prevent GDP/GTP exchange and arrest ribosomal translocation, causing ribosomal stalling and the inhibition of protein synthesis critical for cancer cell survival [51]. Beyond translational arrest, didemnins orchestrate a multimodal antitumor response through redox-mediated apoptosis and microenvironment disruption [30]. By inducing sustained oxidative stress via glutathione depletion, plitidepsin activate JNK/p38 MAPK pathways while simultaneously oxidizing critical cysteine residues (Cys259/Cys276) in MKP-1 to inactivate its phosphatase activity, which creates a feed-forward loop to amplify mitochondrial apoptosis through cytochrome c release [52,53,54,55]. The actin-dependent clustering of death receptors (FAS/CD95, DR5) into lipid rafts establishes membrane-based “apoptotic platforms” that coordinate both extrinsic (caspase-8) and intrinsic (Bid-mediated) death pathways [56,57]. Such dual apoptotic induction is complemented by microenvironment-targeted effects, including the suppression of VEGF/VEGFR-1 signaling and the disruption of protective nurse-like cell interactions, which are particularly evident in hematologic malignancies [58,59,60]. The clinical validation of this mechanism is demonstrated by the Phase III ADMYRE trial of plitidepsin in refractory myeloma, where selective eEF1A2 targeting yielded improved progression-free survival (3.8 vs. 1.9 months) while exhibiting a distinct toxicity profile from proteasome inhibitors and immunomodulatory agents [61,62].

Ongoing studies have revealed that the therapeutic impact of didemnins extends beyond protein synthesis suppression. For example, Losada et al. [63] found that plitidepsin disrupts the binding of eEF1A2 to the pro-apoptotic kinase PKR, which is normally sequestered and inactivated by the elongation factor. By competitively binding to eEF1A2’s C-terminal domain (aa 400-430), plitidepsin displaces PKR, enabling its dimerization and autophosphorylation at Thr446/Thr451. This activates a dual apoptotic cascade through (1) MAPK/NF-κB signaling via eIF2α phosphorylation, and (2) death receptor clustering in lipid rafts. This multipronged attack is further amplified as plitidepsin induces oxidative stress through NADPH oxidase activation, leading to sustained JNK/p38 signaling and MKP-1 degradation [63]. Thus, plitidepsin capitalizes on eEF1A2’s role as a signaling nexus, concurrently dismantling translational, metabolic, and apoptotic evasion mechanisms. A subsequent study showed that the plitidepsin–eEF1A2 interaction induces oxidative stress and ER stress, triggering an atypical unfolded protein response (UPR) marked by eIF2α phosphorylation yet paradoxically accompanied by CHOP degradation [64]. Notably, it simultaneously inhibits autophagy and proteasomal degradation to produce lethal proteotoxic stress. These effects reveal the moonlighting functions of eEF1A2 in stress response coordination, demonstrating how targeting this elongation factor disrupts the proteostatic balance of cancer cells through multiple synergistic pathways. Interestingly, Potts et al. showed that didemnin B features the dual inhibition of palmitoyl-protein thioesterase 1 (PPT1) and eEF1A1, inducing rapid and selective apoptosis in a subset of cancer cells [65]. This polypharmacological action mirrors the targeting of eEF1A2 by plitidepsin but with mechanistic nuances. While both compounds disrupt translation elongation factors (eEF1A1 vs. eEF1A2), didemnin B uniquely couples this with PPT1 inhibition—a lysosomal enzyme critical for palmitoylated protein turnover. The study revealed that the combinatorial suppression of these targets creates a synthetic lethality: translational inhibition depletes short-lived antiapoptotic proteins like Mcl-1, while PPT1 blockade disrupts lysosomal function, collectively overwhelming cellular stress responses. This mechanism parallels plitidepsin’s selectivity for eEF1A2-overexpressing cancers, suggesting that didemnins and their analogs exploit evolutionary vulnerabilities in translation machinery and proteostasis networks. The convergence of these findings indicates the therapeutic potential of targeting elongation factor isoforms in conjunction with context-specific vulnerabilities (e.g., lysosomal dysfunction or redox imbalance) to induce catastrophic stress in malignant cells.

### 2.2. Cytotrienin A and Ansatrienin B

Cytotrienin A (Cyt A) is a natural ansamycin compound derived from *Streptomyces* sp. with potent anticancer activity [66]. It inhibits translation elongation by disrupting the function of eEF1A, thereby stalling actively translating ribosomes and preventing polysome runoff. Unlike peptidyl transferase inhibitors, Cyt A stabilizes the ternary complex (eEF1A–GTP–aminoacyl-tRNA) on the ribosome to impair tRNA release and the subsequent translocation without affecting GTP hydrolysis or ternary complex formation [67]. This mechanism leads to the rapid depletion of short-lived oncoproteins and pro-survival factors critical for cancer cell proliferation and survival. Additionally, Cyt A demonstrates potent antiangiogenic and pro-apoptotic effects by disrupting endothelial cell function and activating dual apoptotic pathways, making it particularly effective against hematologic malignancies [67]. While its translation-inhibitory effects are consistent across cell types, Cyt A induces apoptosis more effectively in leukemia cells, likely due to their heightened dependence on sustained protein synthesis for survival.

In addition to translation inhibition, Cyt A exerts its anticancer effects through a dual signaling mechanism involving the caspase-mediated activation of the MST/Krs kinase pathway and the induction of c-Jun N-terminal kinase (JNK) activity, both of which converge to promote apoptosis in susceptible tumor cells [68]. The compound triggers caspase-3-mediated cleavage of MST1/2 at Asp326/Asn333, generating active 36-kDa fragments (p36 MBP kinase) that phosphorylate myelin basic protein (MBP) at Ser54/Thr98 to drive apoptotic execution [68,69]. Intriguingly, Cyt A also activates JNK independently of the caspase-MST/Krs axis, and this activation similarly depends on ROS but not caspase activity [68]. These coordinated pro-apoptotic mechanisms, combined with its selective modulation of TNF-α signaling through TACE-mediated receptor shedding, establish Cyt A as a unique multifunctional agent against inflammation-associated cancers. Genetic studies reveal that kinase-inactive mutants of MST/Krs or dominant-negative c-Jun each partially inhibit Cyt A-induced apoptosis, while their combined expression nearly abolishes cell death, indicating that both pathways are essential for full apoptotic efficacy [68]. This dual-pathway mechanism indicates the unique capacity of Cyt A to exploit multiple pro-apoptotic routes, offering a strategic advantage for targeting malignancies with diverse resistance mechanisms.

The fact that Cyt A can modulate key cellular signaling pathways is further corroborated by its selective induction of the ectodomain shedding of TNF receptor 1 (TNF-R1) via TNF-α-converting enzyme (TACE) [35]. This process requires the ERK/p38-mediated phosphorylation of TACE at Thr735/Ser819, enhancing its metalloprotease activity to cleave TNF-R1 at Ala76-Val77. By downregulating the cell surface TNF receptor 1, Cyt A selectively impairs TNF-α-induced NF-κB signaling while leaving IL-1α signaling relatively unaffected, as evidenced by its differential inhibition of ICAM-1 expression (IC_50_ 0.8 μM vs. 9.2 μM, respectively). Mechanistically, such specificity arises from the drug’s blockade of TNF-α-triggered IκBα degradation and subsequent NF-κB activation. These effects are nevertheless reversible by TACE inhibitors (TAPI-2) or MAP kinase inhibitors (U0126/SB203580) [35]. The compound’s dual functionality, combining eEF1A-targeted translation inhibition with precision disruption of TNF-α signaling, positions it as a unique anticancer agent against inflammation-driven malignancies.

Ansatrienin B, also known as mycotrienin II, is a structural analog of Cyt A and as such is deemed to work by a similar eEF1A-targeting mechanism that inhibits protein translation [70]. However, it exhibits a distinct mechanism involving the selective suppression of TNF-α-induced inflammatory responses [71]. Specifically, ansatrienin B’s C-13 hydroxyl group forms critical hydrogen bonds with eEF1A’s Arg300/Glu302, enabling the preferential inhibition of TNF-α-stimulated ICAM-1 expression (IC50 = 0.3 μM) over IL-1α-induced responses (IC50 = 3.1 μM). This differential effect correlates with its ability to trigger the TACE-mediated ectodomain shedding of TNF receptor 1 (TNF-R1), thereby attenuating TNF-α/NF-κB signaling, a process dependent on ERK and p38 MAPK activation. It was found that the C-13 hydroxyl group of ansatrienin B is essential for this activity, as demonstrated by the loss of function in a corresponding 13-keto derivative. While its primary action involves eEF1A-mediated translation blockade, the compound’s unique capacity to dysregulate the TNF-R1 surface expression through ribotoxic stress pathways underscores a secondary, context-dependent mechanism that amplifies its anti-inflammatory and potential anticancer effects.

### 2.3. Ternatin

Ternatin is an *N*-methylated cyclic heptapeptide natural product initially identified for its anti-adipogenic activity [72], but its anticancer mechanism remained unclear until Carelli et al. designed synthetic ternatin variants including ternatin-4 with up to 500-fold greater cytotoxicity against cancer cells compared to the parent molecule ternatin [73]. Photoaffinity labeling and competition assays confirmed that these derivatives inhibit protein synthesis by blocking translation elongation. In particular, it selectively targets the eEF1A ternary complex (eEF1A·GTP·aminoacyl-tRNA). Resistance studies identified Ala399 in eEF1A’s Domain III as a critical binding determinant, where mutation to Val (A399V) confers resistance by sterically disrupting ternatin binding, while wild-type eEF1A expression restores sensitivity, genetically validating eEF1A as the direct target. It was observed that structurally unrelated natural products (e.g., didemnin B, cytotrienin A) also compete for eEF1A binding, suggesting a shared functional hotspot for cytotoxic macrocycles [73]. Due to their synthetic accessibility and nanomolar-range potency against diverse cancer cell lines, ternatin derivatives represent promising leads for targeting eEF1A in anticancer drug development.

In a following study, SR-A3 and SS-A3, two epimers of ternatin-4, were designed based on the structure of ternatin-4 [39]. It turned out that SR-A3 is structurally and functionally identical to the natural ternatin, potently inhibiting protein synthesis and cancer cell proliferation by targeting eEF1A. Single-molecule fluorescence resonance energy transfer (smFRET) imaging revealed that SR-A3 exhibits prolonged target residence time and enhanced rebinding kinetics, which was stereospecifically conferred by its (R)-β-hydroxy group, as the removal or epimerization of this moiety significantly reduced activity. In a Myc-driven lymphoma mouse model, the intermittent dosing of SR-A3 significantly reduced tumor burden and improved survival, whereas the non-hydroxylated analog ternatin-4 showed no efficacy. The study not only clarifies the complete stereostructure of A3 but also proposes an evolutionary mechanism by which stereospecific side-chain hydroxylation enhances cyclic peptide binding kinetics. These findings provide new insights into targeting the protein synthesis machinery for cancer therapy. Unlike conventional translation initiation inhibitors, SR-A3 exerts its therapeutic effects through a unique mechanism that prolongs eEF1A stalling on the ribosome and enhances rebinding efficiency, resulting in sustained efficacy [39]. This “residence time pharmacology” paradigm, combined with its efficacy in Myc-driven tumors (which often evade direct Myc-targeting therapies), highlights SR-A3’s clinical potential as a novel protein synthesis inhibitor. Notably, the efficacy of SR-A3 in Myc-driven tumors holds significant clinical relevance, as Myc-dependent cancers typically rely on hyperactivated protein synthesis, yet direct Myc-targeting therapies remain elusive. The study also emphasizes how subtle structural modifications such as β-hydroxylation can optimize the pharmacodynamic properties of natural products, offering a new paradigm for rational drug design.

The unique binding of ternatins to eEF1A was further characterized by structural and dynamic analyses, revealing distinct mechanisms compared to didemnin B [74]. Cryo-EM studies demonstrated that ternatin-4 binds to the same interface between domains I and III of eEF1A as didemnin B, but with a smaller footprint, which likely contributes to its reduced inhibitory efficacy. Unlike didemnin B, however, ternatin-4 induces greater conformational flexibility in eEF1A, particularly in the switch loops of the G domain, which are required for GTP hydrolysis and tRNA accommodation. Such increased dynamics was substantiated by single-molecule FRET (smFRET) experiments, showing the more frequent excursions of the ternary complex to high-FRET states, a result indicative of partial accommodation attempts. In addition, ternatin-4 dissociates from eEF1A approximately 25 times faster than didemnin B, as evidenced by washout experiments, which aligns with its reversible effects on protein synthesis in cellular assays. These findings illustrate how subtle differences in binding kinetics and induced conformational dynamics can lead to divergent pharmacological outcomes, positioning ternatin as a promising scaffold for developing reversible translation inhibitors with potentially reduced toxicity. In an ensuing study, crucial insights were gained into the regulation of eEF1A under the ribosomal stalling conditions induced by ternatin-4, in that there exists a novel ubiquitin-dependent quality control pathway where eEF1A stalling activates the E3 ligases RNF14 and RNF25, leading to eEF1A ubiquitination, with K385 being a candidate site given its location in a solvent-accessible region of Domain III, and subsequent proteasomal degradation, with GCN1 serving as a key sensor that recruits RNF14 to stalled ribosomes while RNF25 primes the pathway through RPS27A ubiquitination [75]. These latest findings have significant implications for developing eEF1A-targeting therapeutics, as the enzyme’s overexpression in cancer cells makes it an attractive drug target, though potential resistance mechanisms involving mutations in eEF1A or the hijacking of the degradation pathway must be considered. The study opens new avenues for research into the physiological triggers of eEF1A stalling, combination therapies targeting both eEF1A and its regulatory ubiquitin ligases, and structural studies to design more specific inhibitors.

### 2.4. Nannocystins

Isolated from the myxobacterium *Nannocystis* sp., nannocystin A and other homologs are potent antiproliferative natural products with strong cytotoxicity against multiple cancer cell lines at nanomolar concentrations, inducing apoptosis rapidly without affecting tubulin or actin dynamics [40,41]. Initially, their mode of action was unclear, but subsequent studies identified eEF1A as the molecular target. Genetic and biochemical evidence confirmed that nannocystin A binds to eEF1A’s Domain III, competitively displacing didemnin B by engaging conserved hydrophobic residues (e.g., Ala399/Val432). Like the preceding eEF1A-targeting inhibitors, nannocystins disrupt tumor growth through multiple pathways. For example, Hou et al. found that nannocystin Ax induces G1 cell cycle arrest by downregulating cyclin D1 expression through eEF1A-mediated inhibition of protein translation, rather than through transcriptional regulation or ubiquitin-proteasomal degradation [42]. This unique mechanism leads to reduced levels of cyclin D1 and its associated cyclin-dependent kinases (CDK4/6), effectively blocking cell cycle progression. Additionally, nannocystin A triggers caspase-independent apoptosis via mitochondrial dysfunction, characterized by the loss of mitochondrial membrane potential, cytochrome c release, and the nuclear translocation of apoptosis-inducing factor (AIF). It was shown that these anticancer effects are p53-independent, suggesting potential therapeutic value even in p53-mutated cancers. In vivo studies using zebrafish xenograft models have demonstrated that nannocystin A significantly suppresses tumor growth at nanomolar concentrations, with an efficacy comparable to the conventional chemotherapeutic agent cisplatin [42]. In a related study, nannocystin Ax was shown to effectively suppress TGF-β1-induced epithelial–mesenchymal transition (EMT), a critical process in cancer metastasis in lung cancer cells [76]. In detail, the drug reversed TGF-β1-induced morphological changes associated with EMT, restoring epithelial characteristics and inhibiting fibroblast-like spindle formation. Mechanistically, it downregulated mesenchymal markers such as N-cadherin and vimentin while upregulating epithelial markers including E-cadherin and claudin-1, indicating the suppression of the EMT program. Furthermore, nannocystin Ax inhibited TGF-β1-driven cell migration and adhesion, which are key steps in metastasis, by inhibiting TGF-β1-induced phosphorylation of Smad2 (Ser465/467) and Smad3 (Ser423/425) and reducing their nuclear translocation, while downregulating TGF-β receptor I (TβRI) expression at the transcriptional level. It also reduced TGF-β receptor I (TβRI) expression at the transcriptional level, suggesting an additional layer of the regulation upstream of Smad activation [76]. These findings highlight nannocystin Ax’s dual role in targeting both proliferative (via eEF1A-mediated cyclin D1 suppression) and metastatic (via EMT inhibition) pathways, supporting it as a promising multitargeted agent against aggressive cancers.

The multitargeting ability of nannocystins was consistent with our recent studies unveiling their complex mode of action that extends beyond the simple inhibition of protein synthesis to include the disruption of critical oncogenic signaling pathways. Our previous work using a coumarin-labeled nannocystin probe demonstrated that these compounds primarily localize to the endoplasmic reticulum (ER), strongly suggesting that ER-bound ribosomes serve as a key site of action [77]. This finding aligns well with the established role of eEF1A in translation elongation at these cellular locations [78]. More recently, we developed a nitroaromatic nannocystin analog (nitro-nannocystin) that exhibited potent anticancer activity both in vitro and in vivo, not only giving superior cytotoxicity compared to its parent compound, nannocystin A0, but also significantly suppressing tumor growth in a patient-derived xenograft (PDX) mouse model without observable toxicity [43]. By means of integrated molecular docking and cellular thermal shift assay (CETSA) approaches, we identified AKT1 as a direct target of nitro-nannocystin, demonstrating its ability to inhibit AKT1 phosphorylation (Thr308/Ser473), while weakly suppressing global protein synthesis (likely via eEF1A inhibition) [43]. This dual mechanism—combining AKT1 pathway blockade with partial translation inhibition—may synergistically enhance antitumor efficacy, though synthetic lethality in PI3K-hyperactivated cancers requires further validation. The hypoxia-sensitive nitroaromatic moiety adds another dimension to this complexity, as its conversion into an active amine intermediate in tumor microenvironments likely contributes to the compound’s in vivo efficacy. These multifaceted properties explain why nitro-nannocystin demonstrates superior potency compared to the traditional single-target inhibitor MK-2206 (which showed higher IC50 values in CRC cells despite targeting AKT1), and accounts for its context-dependent efficacy across various cancer cell lines [43]. Together, these findings establish nannocystins as a unique class of chemotherapeutic agents capable of coordinated disruption at both the translational and signaling network levels.

### 2.5. Metarrestin

Metarrestin is a novel pyrrolopyrimidine derivative [79] discovered through a high-throughput, high-content screening campaign designed to identify compounds that selectively reduce perinucleolar compartment (PNC) prevalence, a marker closely associated with metastatic cancer [80]. The medicinal chemistry optimization of initial hits led to metarrestin, which showed submicromolar potency in reducing PNC prevalence (IC_50_ ~200 nM) while sparing cell viability, achieving a ~38-fold selectivity window compared to cytotoxic effects [45]. Unlike traditional chemotherapeutics such as camptothecin and doxorubicin, which also disrupt the PNC but induce significant cytotoxicity at similar concentrations, metarrestin demonstrated selective antimetastatic activity. Notably, mechanistic studies revealed that metarrestin’s tumor-suppressive effects stem from its unique dual action: (1) disrupting eEF1A2-dependent ribosome biogenesis (via Pol I inhibition) to impair cancer cell proliferation, and (2) reducing metastatic dissemination by selectively targeting PNCs in invasive cells [44]. In multiple rodent cancer models, it effectively suppressed metastasis formation and prolonged survival without evident toxicity. Supported by these compelling preclinical results and a favorable pharmacokinetic profile [81,82,83], metarrestin has advanced into a first-in-human phase I clinical trial to assess its safety and potential as a new therapy specifically targeting metastatic progression in solid tumors [32].

Mechanistically, metarrestin exerts its antimetastatic activity by disrupting nucleolar organization and selectively inhibiting the RNA polymerase I-mediated transcription of ribosomal RNA, a process essential for ribosome biogenesis and cancer cell growth [44]. Treatment with metarrestin induces marked nucleolar segregation and reduces Pol I occupancy at rDNA promoters without broadly affecting RNA polymerase II transcription or causing DNA damage, distinguishing it from classical genotoxic agents. Importantly, the identification of eEF1A2 as a direct binding partner through biotin-conjugated pull-down assays, coupled with the observation that eEF1A2 overexpression rescues metarrestin’s effects, positions it as a key mediator of the compound’s selective action on ribosome biogenesis. In detail, affinity purification studies using a biotin-conjugated analog identified eEF1A2 as a direct binding partner of metarrestin. Functional studies demonstrated that the overexpression of eEF1A2 enhanced PNC prevalence and metastatic burden, while eEF1A2 knockdown phenocopied the nucleolar disruption and rRNA synthesis inhibition observed with metarrestin treatment [44]. This evidence indicates that metarrestin partly exerts its activity through interfering with the non-translational functions of eEF1A2, linking it mechanistically to other small-molecule inhibitors targeting eEF1A such as plitidepsin and didemnin B. However, unlike those compounds, metarrestin appears to selectively modulate nucleolar function and ribosome biogenesis without broadly suppressing global translation, offering a distinct therapeutic strategy for targeting metastatic cancer through eEF1A-dependent pathways. Building on this mechanistic insight, the first potent metarrestin-based proteolysis-targeting chimera (PROTAC) has been disclosed in the literature, exhibiting the effective, dose-dependent degradation of eEF1A2 in multiple cancer cell lines [84]. Overall, these marked advances underscore the versatility of metarrestin and its derivatives as chemical tools to interrogate eEF1A2 biology and as potential therapeutic agents for selectively targeting metastatic processes. The unique ability of metarrestin to disrupt nucleolar function and impair ribosome biogenesis without broadly inhibiting protein synthesis distinguishes it from conventional eEF1A inhibitors, offering a promising strategy to overcome the limitations of cytotoxic chemotherapy. As further preclinical and clinical studies elucidate the safety, efficacy, and optimal applications of metarrestin-based compounds, which also include PROTAC degraders, these agents may emerge as a new class of precision therapeutics for treating eEF1A-driven malignancies and addressing the persistent challenge of metastatic cancer.

### 2.6. BE-43547A_2_

The natural product BE-43547A_2_ was originally isolated from the *Streptomyces* sp. strain A43547. Through total synthesis coupled with biosynthetic gene cluster analysis, Villadsen et al. unambiguously determined its absolute configuration and other congeners, revealing their exceptional hypoxia-selective cytotoxicity against PANC-1 pancreatic cancer cells (IC_50_ ~50 nM) with a selectivity index exceeding 40-fold [85]. Subsequent mechanistic studies demonstrated that BE-43547A_2_ exerts its anticancer effects through a unique mitochondrial-targeted pathway [86]. The compound selectively induces rapid mitochondrial dysfunction in hypoxic conditions (0.5% O_2_), causing cristae disintegration and a loss of membrane potential within 4 h of treatment. This oxygen-dependent cytotoxicity triggers a distinct form of regulated necrotic cell death, accompanied by glutathione depletion and ER stress induction, while remaining resistant to the inhibitors of apoptosis and ferroptosis. Importantly, the anticancer activity requires functional electron transport chain activity, as evidenced by the attenuated effects with ETC inhibitors. These findings were further validated in vivo, where BE-43547A_2_ treatment significantly inhibited tumor growth through necrosis induction, highlighting its potential as a novel therapeutic agent against hypoxic, therapy-resistant tumors [86].

An independent study by Sun et al. reported the exceptional anticancer activity of BE-43547A_2_ toward pancreatic cancer stem cells (PCSCs), a key driver of tumor recurrence and chemoresistance [48]. It was found that BE-43547A_2_ reduces the CD24^+^CD44^+^ESA^+^ PCSC population in PANC-1 cultures by 21-fold at 2 μM, outperforming the clinical candidates AZD7762 (3.4-fold reduction) and LDE225 (1.5-fold). These potent effects reflect BE-43547A_2_’s unique ability to simultaneously target both bulk tumor cells and therapy-resistant PCSC populations through eEF1A1 covalent modification. In tumorsphere formation assays, BE-43547A_2_ at 0.5 μM achieved the 29-fold suppression of PCSC self-renewal, while the in vivo limiting-dilution assays revealed the complete abolition of tumor initiation at 1 μM. These results, coupled with its hypoxia-selective cytotoxicity, indicate BE-43547A_2_ as a dual-action agent capable of eradicating both hypoxic tumor cells and therapy-resistant PCSCs. Using a chemical proteomics approach with a designed activity-based probe, Sun et al. identified eEF1A1 as the direct target, demonstrating the covalent modification of Cys234 through mass spectrometry and site-directed mutagenesis [49]. This interaction was shown to suppress PCSC characteristics by reducing relevant biomarkers (CD133, ALDH1A1, SOX2) and impairing tumorsphere formation—effects that were abolished by Cys234 mutation. The study established a direct correlation between eEF1A1 covalent modification and the disruption of its protumorigenic functions in protein synthesis and PCSC maintenance.

A latest investigation into its molecular mechanism found that the hypoxia-selective BE-43547A_2_ exerts its anti-tumor effects through a sophisticated regulation of the HIF1α-eEF1A1-FoxO1-JAK/STAT3 signaling axis [87]. Under hypoxic conditions (0.5% O_2_), where HIF1α upregulates eEF1A1 expression by 3.8-fold compared to normoxia, BE-43547A_2_ binding disrupts its interaction with FoxO1 as evidenced by co-immunoprecipitation assays, with a consequence of triggering a 3–5-fold increase in FoxO1 nuclear translocation. Nuclear FoxO1 acts as a transcriptional repressor to downregulate JAK1/JAK2 expression, leading to reduced STAT3 phosphorylation at Tyr705, as demonstrated by Western blot and immunohistochemistry, which effectively disrupts a key survival pathway in hypoxic tumor cells [87]. The hypoxia-selectivity was concluded to arise from three coordinated mechanisms: (1) HIF1α-mediated eEF1A1 upregulation; (2) enhanced eEF1A1-FoxO1 complex formation in hypoxia; and (3) oxygen-dependent activation of BE-43547A_2_. This comprehensive mechanism reveals how BE-43547A_2_ precisely targets the hypoxic tumor microenvironment through the multi-level regulation of the eEF1A1-FoxO1-JAK/STAT3 signaling cascade.

## 3. Conclusions

The growing body of research on eEF1A-targeting anticancer agents indicates the therapeutic potential of modulating this critical translation elongation factor in cancer treatment [14]. As discussed above in the preceding Section 2, these targeted inhibitors such as didemnin, plitidepsin, and nannocystins not only disrupt protein synthesis but also interfere with key oncogenic pathways including PI3K-AKT, JAK/STAT3, and TGF-β/Smad signaling, thereby inducing apoptosis, cell cycle arrest, and the suppression of metastasis. Their detailed molecular mechanisms of action are summarized in Table 3. Notably, the anticancer mechanisms of eEF1A inhibitors not listed in this table remain poorly characterized. Recent preclinical studies have further demonstrated that eEF1A1 degradation via the NEDD4L-mediated ubiquitin–proteasome pathway suppresses tumor angiogenesis by inhibiting endothelial cell proliferation and migration, highlighting its therapeutic potential in antiangiogenic therapy [88]. It was also noteworthy that a recent preclinical study demonstrated that EEF1A2 knockdown suppresses lung cancer brain metastasis by inhibiting the BCL10/NFκB pathway and reversing EMT [89], further validating the therapeutic potential of targeting eEF1A isoforms in advanced cancers. Elucidating these mechanisms will provide deeper insights into eEF1A’s critical role in tumorigenesis and cancer progression.

Clinical successes, that is, the approval of plitidepsin for multiple myeloma [31] and the ongoing development of metarrestin for metastatic solid tumors [32], validate eEF1A as a viable drug target. However, challenges remain in optimizing isoform selectivity, minimizing off-target effects, and overcoming potential resistance mechanisms. In particular, the presence of two isoforms, namely eEF1A1 and eEF1A2, presents potential opportunities for targeted therapy development. While these isoforms share 92% sequence identity and conserved core functions in translation elongation, key structural and functional differences critically impact inhibitor design. First, their differential expression patterns, with eEF1A1 being ubiquitously expressed and eEF1A2 restricted to neurons, muscle, and more frequently overexpressed in cancers, suggest that isoform-specific inhibitors could achieve better therapeutic windows. Second, unique structural features like eEF1A2’s Glu300-Asp301 motif (versus eEF1A1’s Cys234) create distinct binding pockets that could be exploited for selective targeting. Third, their divergent post-translational modification landscapes (e.g., eEF1A2’s PE conjugation at Glu301/Glu374 versus eEF1A1’s Cys234-dependent oligomerization) may affect drug binding and resistance mechanisms. Therefore, targeting these differences holds significant promise in that selective eEF1A2 inhibition could treat the neurological disorders caused by EEF1A2 mutations while sparing normal tissues, whereas eEF1A1-specific compounds might address cancers with oxidative stress vulnerabilities.

Future directions should focus on elucidating the structural determinants of eEF1A–inhibitor interactions to guide rational drug design, as well as exploring combination therapies that synergize with existing chemotherapeutics or immunotherapies. The mechanistic insights discussed in this review, particularly the distinct pathways affected by different eEF1A inhibitors (e.g., JAK/STAT3 modulation by BE-43547A_2_, EMT inhibition by nannocystins), provide a strong foundation for developing next-generation combination strategies that simultaneously target translation machinery and critical oncogenic signaling nodes. Importantly, the emergence of resistance-conferring mutations (e.g., eEF1A2-G70S) highlights the need to develop next-generation inhibitors targeting mutant isoforms. Moreover, the development of predictive biomarkers—such as eEF1A overexpression or specific mutational profiles—could enable patient stratification for the precision oncology applications. These mutation profiles may also reveal new therapeutic vulnerabilities for targeted intervention. As our understanding of eEF1A’s moonlighting functions in cancer progression deepens, novel inhibitors with improved efficacy and safety profiles may emerge, including mutation-adapted compounds, offering new hope for patients with aggressive or treatment-resistant malignancies.

## Figures and Tables

**Table 1:** Comparative analysis of eEF1A1 and eEF1A2 isoforms.

Feature	eEF1A1	eEF1A2
Gene Location	Chromosome 6 (human) Chromosome 9 (mouse)	Chromosome 20 (human) Chromosome 2 (mouse)
Expression Pattern	Ubiquitous; dominant embryonic isoform	Neuron/muscle/cardiomyocyte-specific; adult isoform; overexpressed in cancers
Sequence Identity	92% identity, 98% similarity to eEF1A2	More conserved across species than eEF1A1
Key Structural Variations	Asn197 (faster GDP release) Cys234 (oxidation-sensitive oligomerization)	His197 (7-fold slower GDP release) Thr234 (no Cys-like function) Glu300-Asp301 motif (PE modification sites)
PTM Profiles	Cys234-mediated oxidative oligomerization Unique acetylation/methylation (e.g., Lys55)	PE conjugation at Glu301/Glu374 (membrane anchoring) Distinct phosphorylation (e.g., Ser21)
Dimerization Interface	Gly70-independent	Gly70 and Ile71 critical (G70S mutation disrupts dimerization)
Disease Associations	Overexpression linked to cancer progression	Mutations (e.g., G70S, P333L) cause neurodevelopmental disorders and cardiomyopathy
Subcellular Localization	Cytosolic, perinuclear, F-actin bound	Plasma membrane/ER anchored via PE modifications
Therapeutic Potential	Targeting oxidative stress vulnerabilities (e.g., via Cys234)	Neurological disorders (mutation-specific inhibitors) and cancers (membrane-localized targets)

**Table 2:** Anticancer activity and development status of eEF1A-targeting small-molecule agents.

Compound	Anticancer Activity and Development Status	References
Didemnins	Potent against human cancer cell lines from different tissues In vivo validated in several preclinical models Didemnin B and plitidepsin studied in multiple clinical trials Plitidepsin approved in Australia for relapsed/refractory multiple myeloma	[30,31,33]
Tamandarins	Potent against human cancer cell lines from different tissues	[33]
Cytotrienin A	Potent against human leukemia HL-60 cells (IC50 = 7.7 nM) and lung carcinoma A549 cells (IC50 = 0.1 μM)	[34,35]
Ansatrienin B	Potent against several human pancreatic cancer cell lines (IC_50_ 0.17–1.69 μM)	[36]
Narciclasine	Potent against several human melanoma cell lines (IC50~40 nM) In vivo validated in a mice xenograft model of brain metastatic melanoma	[37]
Synthetic flavonoids	Potent against human breast cancer cell lines (IC_50_ 1–50 μM for MDA-MB231)	[38]
Ternatins	Potent against human colorectal carcinoma HCT-116 cells (IC50 = 4.6 nM) In vivo validated in an aggressive Myc-driven mouse lymphoma model	[39]
Nannocystins	Potent against 472 cancer cell lines (IC50 5–500 nM) Nannocystin A validated in an HCT-116-derived xenograft zebrafish model Nitro-nannocystin validated in a patient-derived xenograft mouse model	[40,41,42,43]
Metarrestin	Excellent antimetastatic selectivity over cytotoxicity (cytotoxicity IC50/PNC reduction IC50 = 38.3) In vivo validated in several preclinical models Currently in a phase I clinical trial for the treatment of metastatic solid tumors	[44,45]
Synthetic quinolinones	Potent against human cancer cell lines from different tissues (IC_50_ range, 0.56–50 μM)	[46]
Cordyheptapeptide A	Potent against human colorectal carcinoma HCT-116 cells (IC_50_ = 0.2 μM)	[47]
BE-43547A_2_	Potent against human pancreatic carcinoma PANC-1 cells (IC50 = 0.87 μM) Remarkable hypoxia selective toxicity against human leukemia K562 cells and breast carcinoma MCF-7 cells (selective index = 28 and 79, respectively) Selectively targets pancreatic cancer stem cells (PCSCs) In vivo validated in a pancreatic cancer xenograft mouse model	[48,49]

**Table 3:** Molecular mechanisms of eEF1A-targeting small-molecule agents beyond protein synthesis inhibition.

Compound	Molecular Mechanism of Action
Didemnins	Plitidepsin triggers mitochondrial apoptosis by depleting glutathione, sustaining JNK/p38 activation, and suppressing MKP-1, creating a feed-forward loop for cytochrome c release [52,53,54,55]. Plitidepsin triggers the actin-driven clustering of death receptors (FAS/CD95, DR5) in lipid rafts forms apoptotic platforms that integrate extrinsic (caspase-8) and intrinsic (Bid-mediated) apoptosis pathways [56,57]. Plitidepsin modulates VEGF/VEGFR-1 suppression and disruption of protective stromal interactions, particularly in hematologic malignancies [58,59,60]. Plitidepsin disrupts eEF1A2-PKR interaction, releasing PKR to trigger apoptosis via JNK/p38 MAPK and NF-κB pathways while suppressing pro-survival signals [63]. Plitidepsin induces ER stress while inhibiting autophagy, leading to proteotoxic stress and apoptosis in cancer cells [64]. Didemnin B induces apoptosis by the dual inhibition of PPT1 and eEF1A1, leading to Mcl-1 depletion, REDD1 loss, and mTORC1 activation in sensitive cancer cells [65].
Triene-ansamycins	Cyt A triggers apoptosis via ROS-dependent activation of caspase-3, leading to the proteolytic cleavage of MST/Krs kinases and concurrent JNK activation [68,69]. Cyt A suppresses TNF-α-induced ICAM-1 expression by activating ERK and p38 MAP kinase to trigger TACE-mediated ectodomain shedding of TNF receptor 1, thereby disrupting NF-κB signaling [35]. Ansatrienin B inhibits TNF-α-induced ICAM-1 expression by blocking translation through ribotoxic stress response [71].
Ternatin-4	SR-A3 exhibits the stereospecific enhancement of binding kinetics to eEF1A through prolonged residence time and increased rebinding, leading to potent antitumor activity [39]. Ternatin-4 induces eEF1A stalling, activating an RNF14/RNF25-GCN1 ubiquitin-dependent quality control pathway to clear dysfunctional translation factors [75].
Nannocystins	Nannocystin Ax induces G1 cell cycle arrest and caspase-independent apoptosis in colon cancer by targeting eEF1A, leading to cyclin D1 downregulation and mitochondrial-mediated cell death [42]. Nannocystin Ax inhibits TGF-β1-induced epithelial–mesenchymal transition (EMT), adhesion, and migration in lung cancer cells by suppressing Smad2/3 phosphorylation, nuclear translocation, and downregulating TGF-β receptor type I (TβRI) expression [76]. Nitro nannocystin targets AKT1 to inhibit its kinase activity, suppressing the PI3K-AKT signaling pathway, and inducing cell cycle arrest, apoptosis, and cellular senescence [43].
Metarrestin	Metarrestin inhibits metastasis by disrupting the perinucleolar compartment (PNC) and blocking RNA polymerase I transcription via eEF1A2 interaction [44].
BE-43547A_2_	APD-containing cyclolipodepsipeptides like BE-43547A2 selectively induce mitochondrial dysfunction and necrotic cell death in hypoxic cancer cells [86]. BE-43547A2 selectively targets eEF1A1 in hypoxic pancreatic cancer cells, disrupting its interaction with FoxO1 to induce nuclear translocation of FoxO1 and inhibit the JAK/STAT3 signaling pathway [87].
